# An analysis of the mediating effect of presenteeism between job crafting and organizational silencing in junior nurses: a cross sectional study

**DOI:** 10.3389/fpsyg.2025.1611392

**Published:** 2025-08-15

**Authors:** Na Yang, Dandan Wang, Chen Wei, Jingwen Wang, Liping Yuan

**Affiliations:** ^1^School of Nursing, Wannan Medical College, Wuhu, China; ^2^Nursing Department, Yijishan Hospital of Wannan Medical College, Wuhu, China

**Keywords:** junior nurses, presenteeism, job crafting, organizational silence, mediating effect

## Abstract

**Background:**

Organizational silence is prevalent in the healthcare industry, especially among junior nurses likelier to remain silent on work issues due to their lack of experience and weak voice. This negative behavior not only affects the efficiency of team communication but may also reduce the quality of care. At the same time, presenteeism (working with illness or inefficiency) is becoming increasingly prominent in the nurse population, further exacerbating burnout and organizational silence. Although research suggests that job crafting improves employee initiative, how it inhibits organizational silence by reducing presenteeism is unclear. Therefore, it is important to explore the relationship between the three to optimize nursing management strategies and enhance nurses’ occupational health.

**Objective:**

To explore the mediating effect of presenteeism between organizational silencing and job crafting in junior nurses, to provide intervention targets for clinical nursing management, to reduce organizational silencing, and to optimize nurses’ occupational behavior.

**Methods:**

This study adopted a cross-sectional survey design and strictly followed the STROBE (Strengthening the Reporting of Observational Studies in Epidemiology) guidelines to ensure transparency in research methods and completeness in reporting. A convenience sample of 170 junior nurses (with ≤5 years of clinical experience) was selected from a tertiary hospital in Wuhu City, Anhui Province, and data were collected using a structured questionnaire, and questionnaires were administered using the General Information Questionnaire, Stanford Presenteeism Scales (SPS-6), Job Crafting Questionnaire (JCQ), and Nurse organizational silence assessment questionnaire (NOSAQ). Relationships between variables were clarified by Pearson correlation analysis and the mediating effect of presenteeism was tested by Bootstrap method.

**Results:**

The total score of presenteeism was (15.71 ± 5.65), which was moderately high; the total score of Job crafting (69.35 ± 12.28) showed that the nurses‘ability to proactively restructure their work needed to be improved; and the total score of organizational silence (57.27 ± 14.25) showed that the nurses’ tendency to negatively avoid organizational issues was more obvious. Correlation analysis showed that Job crafting was significantly negatively correlated with organizational silence (*r* = −0.671, *p* < 0.01) and presenteeism (*r* = −0.708, *p* < 0.01); organizational silence was significantly positively correlated with presenteeism (*r* = 0.743, p < 0.01). Mediation effect analyses indicated that presenteeism partially mediated the relationship between job crafting and organizational silence, with a mediation effect value of 47% of the total effect.

**Conclusion:**

Presenteeism is a significant mediating pathway for junior nurses’ Job crafting to influence organizational silence. Nursing managers can improve the status quo by intervening in two pathways: on the one hand, directly improving nurses’ job reinvention ability (e.g., empowering participation in decision-making, optimizing task design), and on the other hand, reducing presenteeism (e.g., improving the sick leave system, reducing work pressure), which can effectively reduce the incidence of organizational silencing, and promote team communication and organizational effectiveness. The findings provide a theoretical basis and practical direction for the development of targeted management strategies.

## 1 Introduction

The organizational silence among nursing staff has become a significant factor influencing the quality of healthcare services and patient safety. The World Health Organization defines registered nurses with ≤5 years of clinical experience as junior nurses (World Health Organization, 2020). As the new generation of the nursing workforce, their professional behavior not only impacts their personal development but also directly affects the stability of nursing teams and healthcare safety. However, current research primarily focuses on senior nurses, and systematic studies on the phenomenon of organizational silence among junior nurses remain scarce. This study innovatively constructs a causal pathway model linking Job crafting-Presenteeism-Organizational silence, thereby revealing the formation mechanism of organizational silence among junior nurses for the first time. The findings will provide a theoretical basis for nursing managers to develop targeted intervention strategies, holding significant practical value for improving the nursing work environment and enhancing team effectiveness.

The concept of organizational silence was first proposed by Morrison and Milliken (2000), who described it as a phenomenon of collective silence culture at the organizational level. Subsequently, Pinder and Harlos (2001) emphasized individual silence behavior. Organizational Silence refers to the behavior of employees who hold opinions or suggestions for improving the organization, but choose to keep their views for various reasons (Yalçın et al., 2022). According to reports, 91.2% of nurses have experienced organizational silence, and 61.6% of nurses choose to remain silent when faced with important issues (Kaya and Eskin Bacaksiz, 2022). In nursing, organizational silence not only weakens nurses’ sense of self-worth and professional identity but also may lead to decreased job satisfaction, burnout, and a tendency to leave (Yağar and Dökme, 2023); in the long term, it may inhibit team innovation and reduce organizational effectiveness (Morrison and Milliken, 2000). Furthermore, nurses’ levels of organizational silence is related to their intention to leave, work engagement, and work performance levels (Yağar and Dökme, 2023). However, there is still a lack of research on organizational silence among junior nurses, particularly as the characteristics of their work may cause their silence to have different causes and consequences, which requires further exploration.

Presenteeism is the behavior of an employee who insists on working despite being ill or unwell (Chapman, 2005). Organizations affected by presenteeism will face reduced productivity and a lack of innovation. A person’s physical presence and problems in the workplace will reduce their work efficiency, as evidence shows that presenteeism reduces productivity by almost four times as much as absenteeism (Mohammadi et al., 2021). This phenomenon is particularly prevalent in the nursing profession, as many as 52.6% of American nurses reported difficulty concentrating on their work at some point during the past 4 weeks. Additionally, the situation in Swedish hospitals is also noteworthy, with a 49% incidence rate of attendance issues among registered nurses and a 47% incidence rate among assistant nurses. In Portugal, this figure stands at 55% (Jianlan et al., 2024). And studies have shown that presenteeism among nurses in China is as high as 77.10% (Yang, 2020). Presenteeism not only harms the physical and mental health of nurses but also reduces their work concentration and communication efficiency, affecting the quality of care (Rainbow, 2019). In addition, it was noted that presenteeism was significantly associated with nurses’ occupational stress and tendency to leave the profession, which may further exacerbate organizational silence (Chun and Hwang, 2019).

Job Crafting (JC) is the behavior of employees who actively adjust their work tasks, interpersonal interactions, and cognitive patterns to optimize their work experience (Yepes-Baldó et al., 2018). Job crafting is a process whereby nurses proactively optimize their work resources, improve their skills, enhance team collaboration, and adjust their job demands. This process not only enhances nurses’ sense of professional fulfillment but also improves the quality of nursing services and team effectiveness (Rodríguez-García et al., 2024). The literature indicates that Job crafting is a powerful strategy that nurses can utilize to optimize their functionality in the workplace and promote alignment between individuals and organizations. Recent studies have shown that job crafting is associated with nurses’ work-life quality (Tawfik et al., 2025), job satisfaction (Yeo and Ha, 2024), perceived health (Zhang X. et al., 2024), organizational identification (Khalifa et al., 2023), and so on. The evidence provided by researchers indicates that high levels of “job crafting” not only improve nurses’ work efficiency and reduce error rates, but also enhance their work engagement (Zhang H. L. et al., 2024), which may have an inhibitory effect on organizational silence. A previous study found that surgical residents with severe intentions to leave demonstrated lower levels of job crafting skills and work engagement (Dominguez et al., 2018). However, to date, no studies have explored the relationship between job crafting and organizational silence among junior nurses.

Therefore, this paper aims to achieve two breakthroughs: First, to construct a theoretical model of job crafting-presenteeism-organizational silence applies to novice nurses; to explore the mediating role of presenteeism between job crafting and organizational silence, to provide nursing managers with more scientific management measures, optimizing the working environment for nursing staff, alleviating the phenomenon of organizational silence among nurses, exploring practical measures to reduce organizational silence among novice nurses, and providing theoretical support and reference for nursing managers are responsible for developing training plans for novice nurses.

Based on the Job Demands-Resources Theory (JD-R), when junior nurses lack job crafting behavior, which leads to a persistent shortage of job resource (such as limited autonomy and insufficient social support), thereby exacerbating work stress and emotional exhaustion. This high demand resources imbalance prompts nurses to persist in working even when physically unwell presenteeism, while reduced psychological safety makes them more likely to remain silent (organizational silence). This theoretical framework provides a systematic explanation for the mechanisms underlying the job crafting-presenteeism-organizational silence relationship.

**Figure 1:**
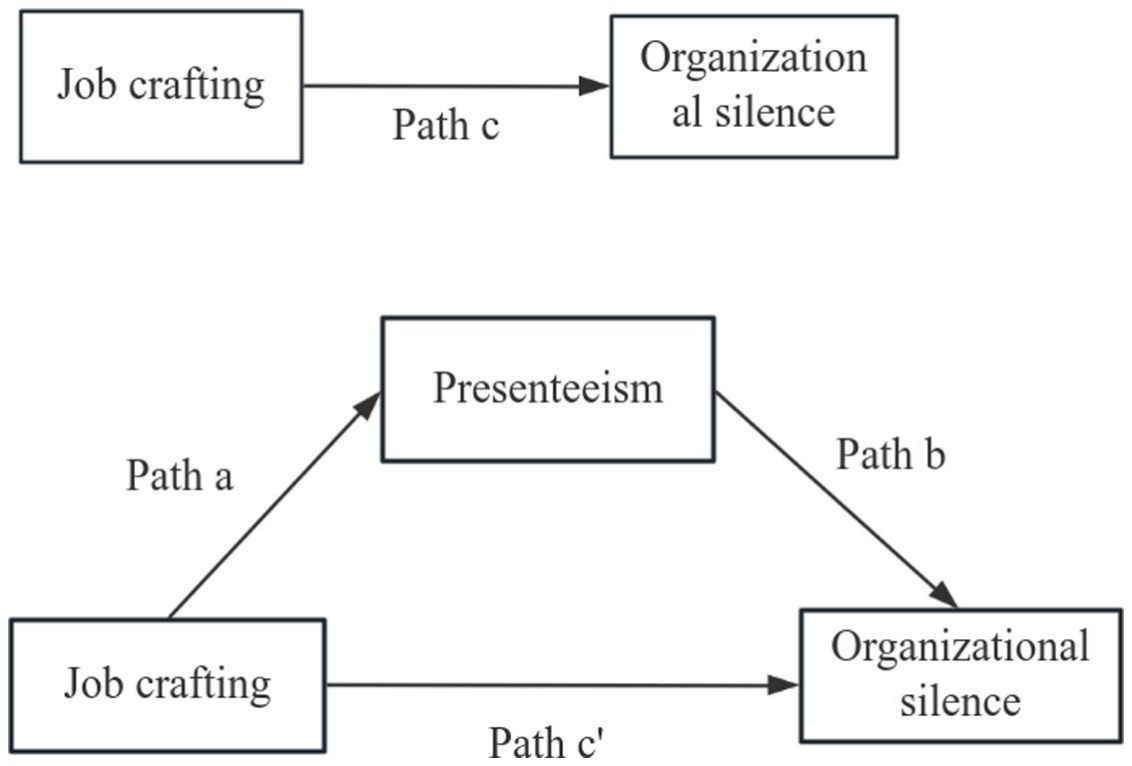
Proposed models that investigate the mediating effect of presenteeism in the association between job crafting and organizational silence.

Based on the above findings, this study proposes four hypotheses (Figure 1).

*Hypothesis 1*: Presenteeism is positively correlated with organizational silence.

*Hypothesis 2*: Job crafting is negatively correlated with organizational silence.

*Hypothesis 3*: Job crafting is negatively correlated with presenteeism.

*Hypothesis 4*: Among junior nurses, Presenteeism plays a mediating role in job crafting and organizational silence.

## 2 Materials and methods

### 2.1 Design

This study strictly adhered to the STROBE (Strengthening the Reporting of Observational Studies in Epidemiology) (Vandenbroucke et al., 2007) guidelines and employed a cross-sectional study design. A tertiary hospital in Wuhu City (with 1,500 nurses) was selected as the study site. As a regional medical center, the hospital’s nursing staff structure and work patterns are representative of those found in similar hospitals across the nation. This single-center design helps to control for institutional-level confounding variables such as hospital culture and shift scheduling, thereby more clearly revealing the interactions between variables.

Convenience sampling was used. A list of nurses who met the inclusion criteria were obtained from the hospital nursing department, and 170 survey subjects were selected. In July–August 2024, 170 junior nurses from a tertiary hospital in Wuhu City, Anhui Province were selected for the study and investigation.

#### 2.1.1 Inclusion criteria

① Hold a valid nursing license and be registered; ② Have 1–5 years of work experience; ③ Currently working on the clinical frontline; ④ Able to independently read, understand, and complete an electronic questionnaire; ⑤ Possess stable internet access and be proficient in using online survey platforms such as Wenjuanxing; ⑥ Voluntarily participate in this study, etc.

#### 2.1.2 Exclusion criteria

① Senior nurses, internship nurses, advanced training nurses; ② those who refused to cooperate with this survey.

According to the STROBE guidelines for reporting sample size requirements, the sample size for this study was calculated as 170 cases based on the cross-sectional study formula. It was calculated using a 95% confidence level (*Z* = 1.96), an expected positive rate of 50%, an allowable error of 7.5%, and a design effect value of 1.5. Although corrected for 153 cases after an overall correction of 1,500, 170 cases, representing 11.3% of all nurses in the hospital, were finalized, taking into account the 90% questionnaire return rate. The sample size calculation process is completely transparent and complies with the STROBE guidelines for methodological reporting.

### 2.2 Instruments

#### 2.2.1 General information questionnaire

The general information questionnaire was designed by the investigator’s literature review as well as group discussion, including gender, age, department, average number of night shifts in a month, education, personnel relationship, title, work scheduling satisfaction, income satisfaction, and reasons for choosing nursing work of the junior nurses.

#### 2.2.2 Job crafting questionnaire (JCQ)

The Job Crafting Questionnaire was developed by Dvorak (2014) and translated into Chinese by Shixiao (2019). It consists of three dimensions: task restructuring (7 items), cognitive restructuring (7 items), and relational restructuring (7 items), totaling 21 items. It uses a 5-point Likert scale, ranging from ‘strongly disagree’ to ‘strongly agree,’ scored from 1 to 5 points, respectively. The scale’s score range is 21 to 105 points, with higher total scores indicating higher levels of work restructuring. In this study, the Cronbach’s *α* coefficient for this scale was 0.951. The Cronbach’s α coefficient for the scale in this study was 0.743.

#### 2.2.3 Nurse organizational silence assessment questionnaire (NOSAQ)

The Nurse Silence Measurement Questionnaire, developed by Yang and Yang (2017) and adapted from Zheng Xiaotao’s Employee Silence Behavior Survey Questionnaire was used to assess the level of organizational silence behavior among nurses in China. The questionnaire comprises 20 items, including negative silence (6 items), defensive silence (6 items), indifferent silence (4 items), and prosocial silence (4 items). A 5-point Likert scale was used, with 1 being the lowest score and 5 the highest, resulting in a total score range of 20–100. Higher scores indicate more severe levels of silent behavior. The overall Cronbach’s *α* coefficient for the questionnaire was 0.918, while the Cronbach’s α coefficient for this study was 0.751.

#### 2.2.4 Stanford presenteeism behavior scale (SPS-6)

The Stanford Presenteeism Scales (SPS-6) were developed by Koopman et al. (2022), consisting of 6 items. Chinese scholars (Zhao et al., 2010) adapted and revised the scale into Chinese, creating the Chinese version of the SPS-6, which includes two dimensions: work limitations and work energy, comprising 6 items in total. The total score ranges from 6 to 30 points, with higher scores indicating more severe productivity losses due to working while ill. The Cronbach’s *α* coefficient is 0.76.

### 2.3 Data collection method

Data was collected through an online survey platform^1^ using electronic questionnaires that included informed consent forms. Participants who agreed to participate in the study answered all questions according to the instructions, with the survey taking approximately 10–15 minutes to complete. Questionnaire completion permissions were set so that each IP address could only submit one valid response. Each item was designated as a required field to prevent omissions, and questionnaires with obvious filling patterns were excluded. After completing the questionnaire, each participant received a random compensation of 5 Chinese yuan (CNY).

### 2.4 Ethical consideration

This study was approved by the Ethics Committee of Wannan Medical College. The purpose and methodology of the survey were explained to the participants by the researcher before the start of data collection, and we emphasized the principles of anonymous participation and voluntary withdrawal, and ensured that participants understood that they had the right to withdraw from the study at any time without any repercussions. All subjects who agreed to participate were allowed to access the questionnaire and complete it only after signing an informed consent form. To safeguard participants’ privacy and data security, the questionnaires in this study did not collect any information that might reveal participants’ identity (e.g., name, school, ID number, etc.)

### 2.5 Statistical methods

Data analysis was conducted using IBM SPSS Statistics 27.0. Descriptive statistics, including means, standard deviations, frequencies, and percentages, were used to describe participants’ sociodemographic characteristics and measurement scores. Analysis of variance (ANOVA) and t-tests were employed to examine differences in organizational silence among junior nurses across sociodemographic characteristics. Pearson correlation coefficients were calculated to assess the relationships among job crafting, presenteeism, and organizational silence. Multiple linear regression and mediation analysis were conducted using the PROCESS macro (Model 4) (Hayes, 2013). The bootstrapping method used 5,000 resamples of the data to generate 95% bias-corrected confidence intervals. If the confidence interval did not include zero, the effect was considered statistically significant. In this study, a two-tailed *p* < 0.05 was considered statistically significant.

## 3 Results

### 3.1 Basic information on junior nurses

Among the 170 valid questionnaires, 20.6% were completed by males and 79.4% by females; 55.3% were aged 29–25, and 44.7% were aged 25–35; 27.6% had an introverted personality, 40.0% had an extroverted personality, and 32.4% had an intermediate personality; the departments they belonged to were internal medicine, surgery, ICU, emergency department, other departments accounted for 21.2, 19.4, 24.1, 12.4, and 22.9%, respectively; for more details, please refer to Table 1.

**Table 1:** Comparison of general information and organizational silence scores among junior nurses with different characteristics (*n* = 170; mean ± SD).

Variables	Junior nurses of organizational silence (*n* = 170)				
	Category	*n* (%)	Organizational silence (mean ± SD)	*F*/*t*	*p*
Gender	Male	35 (20.6%)	51.94 ± 2.43	−0.16	0.877
	Female	135 (79.4%)	52.36 ± 1.22		
Age	20–25	94 (55.3%)	52.05 ± 1.41	−0.22	0.826
	25–35	76 (44.7%)	52.54 ± 1.72		
Character	Introvert	47 (27.6%)	54.23 ± 1.85	1.41	0.248
	Extrovert	68 (40.0%)	50.07 ± 1.70		
	Intermediate	55 (32.4%)	55.31 ± 2.10		
Department	Internal Medicine	36 (21.2%)	51.80 ± 2.55	0.94	0.443
	Surgery	33 (19.4%)	50.85 ± 2.41		
	ICU	41 (24.1%)	55.05 ± 2.37		
	Emergency Medicine	21 (12.4%)	54.57 ± 2.70		
	Other Departments	39 (22.9%)	49.67 ± 2.17		
Average number of night shifts in a month	≤4	95 (55.9%)	50.81 ± 1.34	−1.52	0.133
	5–9	75 (44.1%)	54.90 ± 1.75		
Academic qualifications	Technical secondary school (has obtained a nursing license)	24 (14.1%)	63.42 ± 2.85	16.43	<0.001^***^
	College	41 (24.1%)	58.10 ± 2.07		
	Undergraduate	81 (47.6%)	49.16 ± 1.47		
	Master	24 (14.1%)	41.67 ± 1.38		
Personnel Relationship	Labor dispatch personnel	54 (31.8%)	56.19 ± 1.97	4.00	<0.001^***^
	Contractual	75 (44.1%)	51.75 ± 1.71		
	Regularly on board	41 (24.1%)	48.07 ± 1.82		
Title professional title	Nurse	122 (71.8%)	53.20 ± 1.21	1.26	0.212
	Nurse and above	48 (28.2%)	49.90 ± 2.23		
Shift scheduling in the past year	Very satisfied	23 (13.5%)	41.74 ± 1.57	9.60	<0.001^***^
	Satisfactory	46 (27.1%)	49.07 ± 1.83		
	Average	65 (38.2%)	52.86 ± 1.86		
	Quite dissatisfied	24 (14.1%)	61.71 ± 2.66		
	Dissatisfied	12 (7.1%)	62.67 ± 3.21		
Income in the past year	Very satisfied	35 (20.6%)	43.69 ± 1.45	16.25	<0.001^***^
	Satisfied	35 (20.6%)	48.67 ± 1.86		
	Average	31 (18.2%)	51.32 ± 2.35		
	Quite dissatisfied	21 (12.4%)	57.81 ± 3.25		
	Dissatisfied	29 (17.1%)	66.34 ± 1.82		
Reasons for choosing nursing	Passionate about nursing	52 (30.6%)	45.00 ± 1.49	11.95	<0.001^***^
	Suggested by elders/relatives	60 (35.3%)	53.82 ± 1.75		
	For survival needs or others	58 (34.1%)	57.19 ± 2.01		

*p < 0.05, **p < 0.01, ***p < 0.001, *n*, sample size for each category; %: percentage of the total number of samples (*N* = 170); *M* ± SD: mean ± standard deviation of the Organizational Silence Score; F/t: t-value based on comparisons (for independent *t*-tests) or *F*-value (for analysis of variance); *p*: level of significance (*p* < 0.05 indicates statistical significance).

### 3.2 Presenteeism of junior nurses

The total score of presenteeism of 170 junior nurses was (15.71 ± 5.65), with (9.76 ± 4.19) in the dimension of work constraints and (5.95 ± 2.04) in the dimension of work energy.

### 3.3 Job crafting of junior nurses

The total score of job crafting of 170 junior nurses was (69.35 ± 12.28); the scores of each dimension were (23.91 ± 4.80) for relationship remodeling, (22.80 ± 5.15) for task remodeling, and (22.64 ± 4.46) for cognitive remodeling.

### 3.4 Organizational silence of junior nurses

Total score of organizational silence of 170 junior nurses (57.27 ± 14.25); scores of dimensions Defensive silence (16.38 ± 5.91), Negative silence (16.97 ± 4.87), Indifference silence (8.00 ± 3.12), Pro-social silence (10.92 ± 4.15).

### 3.5 Correlation of junior nurses’ presenteeism, job crafting, and organizational silence

The correlation coefficients for each variable in this study are shown in Table 2. Correlation analysis revealed that presenteeism was significantly positively correlated with organizational silence (*r* = 0.743, *p* < 0.01), presenteeism was significantly negatively correlated with job crafting (*r* = −0.608, *p* < 0.01), job crafting was significantly negatively correlated with organizational silence (*r* = −0.671, *p* < 0.01). These results indicate that job crafting and presenteeism are important factors influencing organizational silence among junior nurses.

**Table 2:** Correlation of presenteeism, job crafting, and organizational silence among junior nurses.

Variables	Presenteeism	Organizational silence	Job crafting
Presenteeism	1		
Organizational silence	0.743**	1	
Job crafting	−0.608**	−0.671**	1

**p < 0.01.

### 3.6 Mediating effect of job crafting between organizational silence and presenteeism in junior nurses

Using the silence score of junior nurses as the dependent variable and statistically significant variables from the univariate analysis as independent variables, a multiple linear regression analysis was conducted. The results showed that educational attainment, shift satisfaction, and income satisfaction were significant factors influencing the silence of junior nurses (*p* < 0.05). Table 3.

**Table 3:** Regression analysis of organizational silence.

Model	Unstandardized coefficients		Standardized coefficients	*t*-value	*p*-value
	*B*	Standard error	Beta		
(Constant)	48.609	4.929		9.862	0.000***
Academic qualifications	−5.261	1.166	−0.331	−4.512	0.000***
Personnel Relationship	0.918	1.327	0.048	0.692	0.490
Shift scheduling in the past year	2.352	1.027	0.179	2.290	0.023*
Income in the past year	2.750	0.912	0.265	3.016	0.003**
Reasons for choosing nursing	0.835	1.285	0.047	0.650	0.517

*p < 0.05, **p < 0.01, ***p < 0.001, *R*^2^ = 0.359; *N* = 170; *F* = 19.93.

The common method bias was assessed using Harman’s single-factor test. The results showed that the number of common factors with eigenvalues greater than 1 was less than 10, and the proportion of variance explained by the first common factor (30.3%) was significantly lower than the critical threshold of 40%.

**Figure 2:**
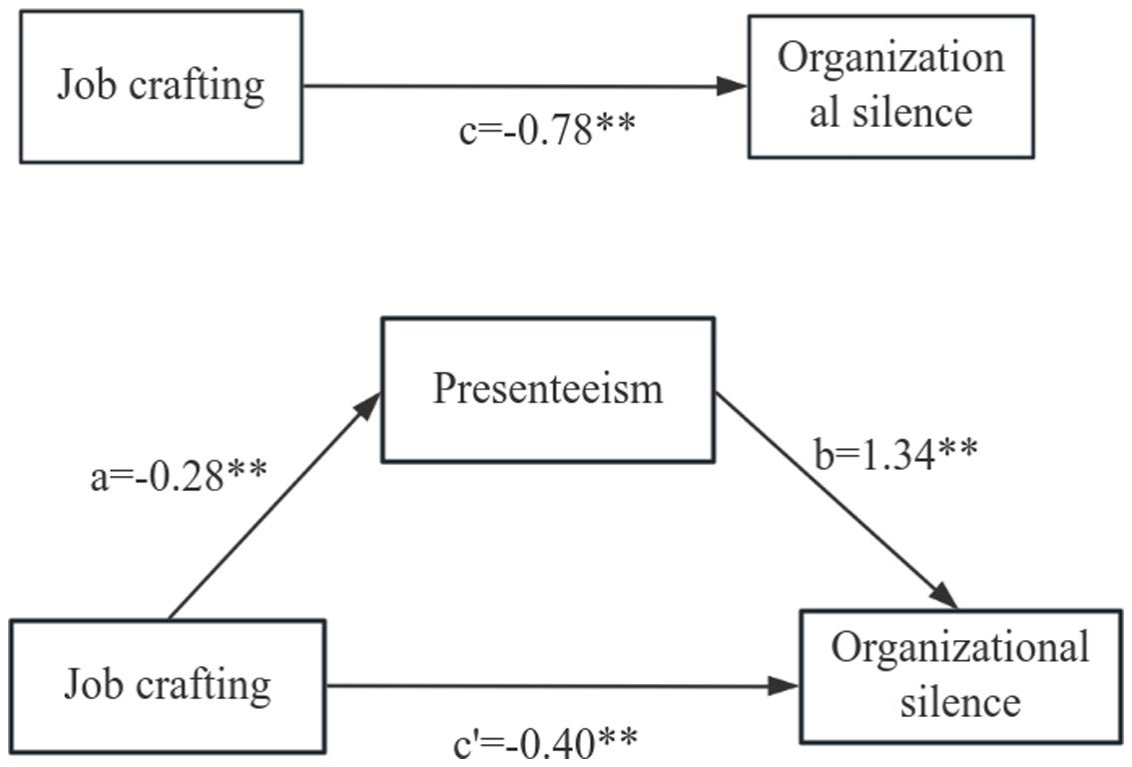
Presenteeism mediated the association between job crafting and organizational silence. a and b are the mediating effects of presenteeism as a mediating variable; c’ is the direct effect; **p < 0.01.

The results showed that the 95% confidence interval of the mediating effect of presenteeism between Job crafting and organizational silence among junior nurses was −0.48 to −0.27 (excluding 0), with all *p*-values <0.01, and the mediating effect was statistically significant, controlling for demographic variables, and the mediating effect of Job crafting and organizational silence was examined, and the results are shown in Table 4. The analysis of the mediating effect of Job crafting between presenteeism and organizational silence among junior nurses is detailed in Table 5. The analysis of the mediating effect is detailed in Table 4. The relationship between the variables is shown in Figure 2.

**Table 4:** Analysis of the mediating effect of presenteeism between the job crafting of junior nurses’ work and organizational silence.

Outcome variables	Predictive variables	Fitness indicator		Significance	
		*R* ^2^	*F*	*t*	*p*
Organizational Silence	Job crafting	0.45	137.55	−11.73	0.00**
Presenteeism	Job crafting	0.37	98.44	–9.92	0.00**
Organizational silence	Presenteeism	0.63	141.35	−5.85	0.00**
	Job crafting			8.96	0.00**

**p < 0.01.

**Table 5:** Total, direct and mediating effects of presenteeism between organizational silencing and job crafting in junior nurses.

Variables	Effect value	Boot SE	95 percent confidence interval (CI)		Relative effect value (%)
			Upper limit	Lower limit	
Total effect	−0.78	0.07	−0.91	−0.65	
Direct effect	−0.40	0.07	−0.54	−0.27	51%
Indirect effect	−0.37	0.05	−0.48	−0.27	47%

Standard errors, upper and lower bounds of 95% confidence intervals for indirect effects estimated using the Bootstrap method.

## 4 Discussion

### 4.1 Analysis of the current state of junior nurses’ presenteeism, job crafting, and organizational silence

#### 4.1.1 Characteristics of the current situation of presenteeism among junior nurses

The results of this study showed that junior nurses had a high level of presenteeism (15.71 ± 5.65 scores), a result that is generally consistent with the findings of Li et al. (2025). It is worth noting that the presenteeism scores in this study were significantly higher than the findings of scholars such as Ping et al. (2023), Jiang et al. (2023), and Shujie et al. (2021) and this difference may have stemmed from different factors such as the working seniority of the study participants and the characteristics of the department. In-depth analyses revealed that the under-allocation of nursing human resources in China [only 2.17 registered nurses per 1,000 population (Liu, 2017)] is an important social factor contributing to the high incidence of presenteeism. Clinical nurses generally face overloaded work pressure, and frequent night shift rotation makes ‘no leave’ a common phenomenon. In addition, the design of the performance appraisal system, which is overly dependent on presenteeism, and the subjective willingness of junior nurses to improve their professional skills by increasing the number of hours they spend on the job, further exacerbate this phenomenon. Of particular interest is the fact that healthcare workers in this study scored particularly highly on the ‘work constraints’ dimension, reflecting the potential impact of working while sick on the quality of care. This finding echoes the findings of Ni et al. (2023), suggesting that nursing managers should improve the current situation by optimizing the allocation of human resources and reforming the performance appraisal system (Zhang H. L. et al., 2024).

#### 4.1.2 Characteristics of the current situation of job crafting among junior nurses

The study data showed that the job reinvention ability of junior nurses was at a medium level, which was consistent with the findings of Siyu et al. (2024), higher than the results of Ghazzawi et al. (2021) (54.42 ± 8.05), but significantly lower than those reported by Zhijie (2023) and Duan et al. (2024). This gap may stem from the following reasons: firstly, insufficient career development support for junior nurses in healthcare organizations, resulting in a lack of necessary job reinvention skills; and secondly, a lack of effective guidance from senior nurses, resulting in a lack of a sense of direction in job reinvention practice (Embree, 2022). Among the three dimensions of job reinvention, relational reinvention performed the best, task reinvention was the second best, and cognitive reinvention was the weakest. The low level of cognitive remodeling reflects systematic deficiencies in the current nursing training system, and the lack of an innovative culture in the work environment inhibits nurses’ willingness to try new approaches. It is recommended that nursing managers establish an incentive mechanism for innovation, incorporate job reinvention ability into the core competency training system for nurses, and at the same time provide professional mentors for junior nurses to provide personalized development guidance.

#### 4.1.3 Characteristics of the current status of organizational silence among junior nurses

This study found that the phenomenon of organizational silence was more serious among junior nurses, a result that was consistent with the findings of Wu et al. (2023), higher than the results of Yang et al. (2022), Lv et al. (2024), and other studies. In-depth analyses revealed that low professional self-confidence and weak organizational status were the main reasons for silence among lower-aged nurses. Notably, the level of organizational silence varied significantly across groups of nurses with different characteristics: nurses with education at the secondary and tertiary levels showed a higher tendency to remain silent, these findings suggest that nursing managers should pay particular attention to the career development needs of nurses with lower educational attainment. They should provide targeted training to enhance their professional capabilities and communication skills, while also establishing a more inclusive organizational culture that encourages nurses at all levels to actively participate in work improvement and decision-making processes. Labor-dispatch nurses had a significantly higher level of silence due to a weak sense of organizational belonging, and nurses who were dissatisfied with their scheduling and income were also more inclined to remain silent. First, dissatisfaction with the shift scheduling system can easily lead to occupational burnout and emotional exhaustion, reducing nurses’ psychological attitude towards participating in organizational suggestion-making. Second, dissatisfaction with income may weaken organizational commitment, leading nurses to believe that suggestion-making will not bring about substantial change. Therefore, it is recommended that nursing managers establish effective feedback channels to allow nursing staff to freely express their opinions on scheduling and income. Scheduling system optimization: analyze the shortcomings of the existing scheduling system and consider introducing flexible scheduling methods to meet employees’ personal needs and work-life balance; ensure transparency in the income structure, explain the compensation policy to employees, and increase their understanding and acceptance of their income. Nursing managers should select relevant and effective assessment tools and develop tiered management plans tailored to different levels of organizational silence to maximize the positive benefits of organizational silence (Wen et al., 2025).

### 4.2 Correlation analysis of job crafting with presenteeism and organizational silence among junior nurses

This study revealed significant patterns of association between Job crafting, presenteeism, and organizational silence through correlation analysis. The results of the study showed that there was a significant positive correlation between presenteeism and organizational silence (*r* = 0.743, *p* < 0.01) (Verification of Hypothesis 1), a finding that supports the hypothesis of a vicious circle of presenteeism-organizational silence. Specifically, when junior nurses are forced or actively choose to work with illness, their physiological and psychological resources continue to be depleted, resulting in the accumulation of negative emotions and decreased work engagement. In this state, nurses often tend to reduce communication with colleagues and managers, and in turn, show more pronounced organizational silencing behaviors. Especially in high-pressure healthcare work environments, junior nurses are more likely to choose to ‘tough it out’ rather than express their situation openly for reasons of occupational safety, a coping strategy that further strengthens their tendency to remain silent. To address this phenomenon, it is recommended that nursing managers should intervene in the areas of human resource allocation and workflow optimization: on the one hand, they should establish a scientific staffing model to ensure sufficient nursing manpower in each department; on the other hand, they should simplify nursing paperwork and reduce unnecessary repetitive labor, to reduce nurses’ workload.

The study also found that Job crafting was significantly negatively correlated with organizational silence (*r* = −0.671, *p* < 0.01) (Verification of Hypothesis 2), this result can be rationalized by self-efficacy theory: when nurses can proactively adjust their work tasks, cognitive patterns, and interpersonal relationships, their sense of control over the work environment significantly increases. This sense of control not only enhanced professional confidence but also reduced the perceived risk of advice, making nurses more willing to share their opinions and suggestions. Nursing managers are advised to promote job reinvention behaviors by: organizing regular job reflection sessions to encourage nurses to share suggestions for improvement; establishing a ‘problem-solution’ oriented communication mechanism to make nurses feel the value of their advice; and incorporating job reinvention skills into the nurses’ career development assessment system (Jingjing et al., 2023).

In addition, there was a significant negative correlation between job reinvention and presenteeism (*r* = −0.608, *p* < 0.01) (Verification of Hypothesis 3). Consistent with the results of Liu et al.’s (2022) research. Nurses with high levels of job crafting competence usually showed stronger intrinsic work motivation and professional autonomy. They are more concerned with the quality of their work and professional growth rather than simply meeting presenteeism requirements. This working attitude enables them to better balance work and health, and they are more inclined to choose reasonable rest rather than forced work when they are not feeling well. To cultivate this positive work attitude, it is recommended that nursing managers: implement participatory management and give nurses appropriate work autonomy; establish a diversified performance evaluation system, weakening the weight of the assessment of simple presenteeism; and carry out regular education on professional values to strengthen nurses’ sense of professional identity.

### 4.3 Analysis of the mediating effect of junior nurses’ presenteeism between job crafting and organizational silence

This study provides insight into the key mechanisms of the role of presenteeism in the relationship between Job crafting and organizational silencing among junior nurses through mediated effects analysis. The findings indicated that presenteeism played an important partial mediating role in the relationship between Job crafting and organizational silence, with a mediating effect size of 47% of the total effect, 95% CI (−0.48, −0.27) (Verification of Hypothesis 4), a significant proportion that has far-reaching theoretical and practical value. The study found that the effect of Job crafting on organizational silencing showed a two-channel mechanism of action: the direct effect accounted for 53% of the total effect, suggesting that Job crafting per se has a dominant role in suppressing organizational silencing; meanwhile, the indirect effect through the reduction of presenteeism accounted for 47% of the total effect, which is significantly higher than that of the mediating effect commonly found in the field of healthcare organizations (typically 30–35%) (Preacher and Kelley, 2011), and this sizable indirect effect suggests us that improving presenteeism is an equally important breakthrough in reducing organizational silence.

In terms of the path of action, the effect of Job crafting on organizational silence presents a dual mechanism: on the one hand, Job crafting can directly reduce the level of organizational silence; on the other hand, it indirectly affects organizational silence through the reduction of presenteeism behaviors. Specifically, when the level of Job crafting increases in junior nurses, their tendency to be presentist decreases accordingly, which in turn reduces the level of organizational silence. This finding echoes the results of a previous study on high-performance work systems (Elliott et al., 2011), suggesting that nurses’ productivity and quality of work can be significantly improved by enhancing their professional identity and Job crafting competencies, which in turn enhances their professional confidence and sense of identity, and ultimately reduces the prevalence of organizational silencing.

An in-depth analysis of the internal mechanism of this mediating effect can be elucidated at the following levels: first, through task remodeling, nurses can rationally prioritize their clinical work and optimize their workflow; second, cognitive remodeling helps nurses deepen their understanding of their responsibilities and competencies, and enhances their sense of mission at work; and further, relational remodeling promotes the establishment of good interpersonal relationships. Together, these reinventing behaviors enabled nurses to better manage themselves, enhance their learning ability, mobilize positive emotions to cope with work challenges, and make effective use of organizational resources. This set of positive changes not only increased productivity, but more importantly reduced the phenomenon of being forced to work with illness (presenteeism), enabling nurses to maintain a better physical and mental state during working hours, which in turn led to a greater willingness to express their views and suggestions, and a reduction in the incidence of organizational silencing.

Based on these findings, the following recommendations are made for nursing management practice: In terms of system construction, it is suggested that hospitals should reform the existing assessment and evaluation system, incorporate the ability to reshape work into the indicators for assessing nurses’ competence, and appropriately reduce the weight of presenteeism rates in performance appraisals, paying more attention to the quality of work and innovative contributions. Meanwhile, a scientific sick leave management system is established, and a reasonable number of paid sick leave days is set to eliminate the economic concerns of nurses working with illness. In terms of talent cultivation, it is recommended to carry out a systematic training program on job reinvention ability, to enhance nurses’ job reinvention skills through case teaching and scenario simulation, and to provide professional mentors for junior nurses to provide personalized development guidance. In terms of environmental optimization, it is recommended that hospitals improve the working conditions of nurses, set up special rest areas, reasonably arrange work intensity, and establish a mental health support system with regular stress assessment and psychological counseling services. In addition, it is recommended that hospitals develop diversified communication channels, including an anonymous suggestion system and regular symposiums, to encourage nurses to actively make suggestions and give substantive feedback and rewards for valuable suggestions. The comprehensive implementation of these measures will help break the vicious circle of ‘low job crafting—high presenteeism—high organizational silence’, and promote the professional development of the nursing team and the improvement of service quality.

## 5 Limitations

Although this study strictly adhered to STROBE guidelines for design and reporting, the following limitations exist: First, the study sample was drawn exclusively from a tertiary hospital in Anhui Province. The characteristics of a single-center sample may be influenced by the regional distribution of medical resources, hospital management culture, and other factors, necessitating caution when generalizing the study results across regions. Future studies could expand the sample size and adopt a multi-center sampling design to enhance the external validity of the results. Second, this study used a questionnaire survey method to collect data, which may be subject to subjective reporting bias due to factors such as respondents’ emotional states and misunderstandings of the questionnaire. It is recommended that future studies combine behavioral observation methods or objective indicators for validation. Furthermore, while cross-sectional study designs can reveal covariate relationships between variables, they cannot infer the causal sequence of ‘Job crafting—presenteeism—organizational silence.’ Future studies may adopt longitudinal study designs to more precisely validate theoretical hypotheses.

## 6 Conclusion

This study contributes to the development of relevant theories in three ways by validating the mediating role of presenteeism in the relationship between job crafting and organizational silence among junior nurses: first, it expands the application boundaries of job crafting theory, revealing the unique mechanism by which healthcare professionals engage in silent behavior due to working while ill in resource-constrained situations; second, it enriches the antecedent research of organizational silence theory, it is the first empirical test of the ‘double-edged sword’ effect of employee proactive behavior on silence; finally, it innovatively constructs a theoretical chain of ‘behavioral restructuring-health depletion-defensive silence,’ providing a new perspective for understanding the special behavioral patterns of junior nursing staff. These findings not only fill the gap in existing research on the behavioral mechanisms of sub-healthy employees in the healthcare industry but also provide a theoretical basis for healthcare institutions to establish early intervention mechanisms and optimize communication channels. System, providing training in Job crafting capabilities, and establishing a tiered feedback mechanism.

Future research may consider exploring the following areas in greater depth: first, the sample source should be expanded to include medical institutions of different levels and in other regions (such as community hospitals and specialized hospitals), and a multi-centre collaborative research design should be adopted to improve the representativeness and generalisability of the results; second, a longitudinal tracking design or randomized controlled trial should be adopted to verify the causal relationship between variables, and objective indicators such as electronic medical records and behavioral observations should be combined with subjective report data for multi-method verification; Additionally, the impact of potential moderating variables such as departmental differences, years of experience, and professional title levels should be thoroughly examined, and the differences in mechanisms of action across various medical settings should be explored; finally, cross-cultural comparative studies should be conducted to assess the applicability of research conclusions across different healthcare systems and cultural contexts, while qualitative interviews and other methods can be employed to deeply explore underlying mechanisms, providing theoretical foundations for developing more targeted intervention measures.

## Data Availability

The data set generated by this study can be obtained from the first author upon reasonable request.

## Footnotes

^1^ https://www.wjx.cn/
